# Most people can stay fit and healthy into old age

**DOI:** 10.2471/BLT.14.030914

**Published:** 2014-09-01

## Abstract

Bruno Vellas tells Fiona Fleck why governments must act now to help older people stay fit and healthy.

Q: How did you become interested in the field of ageing?

A: Geriatric medicine is a relatively new field. It started around 40 to 50 years ago when people with severe dependency turned up in hospital emergency wards. No one knew what to do with them and so geriatric wards were established. I became interested in this field because I felt very strongly that we needed to act at an earlier stage to prevent this happening. I wanted to understand the biology of frailty better to find ways to prevent dependency. Today’s geriatric specialists are rather like oncologists 30 years ago, who only saw patients with late stage cancer. In recent years, we have found ways to prevent cancer, as well as to treat and even cure it.

Q: What is frailty in the clinical sense?

A: There are five criteria, developed by Linda Fried of Columbia University in New York, and these have become standard: involuntary weight loss, sedentary lifestyle with low physical activity, exhaustion, slow gait speed (more than 1 second per metre in a 4 metre test) and muscular weakness. A patient with one of these is considered to be “pre-frail” and a patient with three of them is considered to be “frail”.

Q: How much frailty can be prevented?

A: About 50% of the loss of autonomy in frail older adults, while they are hospitalized, is avoidable. We did a survey at Toulouse University Hospital, the fourth largest hospital in France, with more than 1500 beds and found that during the study period nearly 650 beds were occupied by people aged 70 years and more and that nearly 20% of them lost some autonomy and some functions during their hospitalization. In half of these cases, the cause of functional decline was not related to their medical condition but to the failure of the health-care system to adapt to their needs. About 30% of people in this age group are hospitalized at least once a year – so this is a common problem – and 10% of them will present with avoidable severe disability due to their hospitalization. For example, some could not dress themselves, while others became incontinent or could not walk any more. Health professionals are aware of the need to prevent hospital-acquired infections, but much less of the need to maintain patients’ functions during a stay in hospital. People in good health usually don’t lose their ability to function independently in hospital, but the frail do. We must act now to prevent this needless loss of autonomy.

“About 50% of the loss of autonomy in frail older adults, while they are hospitalized, is avoidable.”

Q: At what stage can frailty be prevented?

A: We have good scientific evidence to show that until a person shows signs of frailty, this process can be reversed. However, once a person can no longer continue his or her basic daily activities, it is often too late. Many epidemiological studies show that frailty is a precondition for dependency, while other studies show that if we intervene at the pre-frail and frail stages, we can return the patient to a stage at which he or she is physically robust or, at least, able to avoid dependency. But first we need to identify the older adults who are pre-frail and frail as early as possible. Then we need to look at the causes. Sometimes these conditions can be related to a disease that has not been diagnosed, for example cardiovascular disease, or a problem with their eye sight or hearing, or even can be related to social isolation. This is the stage at which a multi-domain intervention is often needed.

Q: What is that?

A: A multi-domain intervention means providing a person with support on several levels, including nutrition, physical exercise and social support. We have strong evidence to show that physical exercise has a beneficial effect on many aspects of human biology, from individual cells to the full human body. Health-care services in high and middle-income countries are increasingly confronted with large groups of elderly people with physical impairments and resulting loss of mental and physical functions. That’s why our research centre in Toulouse recently launched the SPRINT clinical trial in collaboration with an academic centre in Rome and industry partners. It has 1500 participants aged over 70 in Europe and aims to find ways to prevent dependency by taking a multi-domain intervention approach with regard to nutrition, physical exercise and new technologies. It is part of the Innovative Medical Initiative and is funded by the European Union. We will also soon have the results of the Multi-Domain Alzheimer’s Preventive Trial (MAPT). 

Q: Can simple tests detect conditions affecting eye sight, such as glaucoma and macular degeneration and impaired hearing? At what stage and how can these be prevented?

A: We need to change medical practice so that it focuses more on maintaining sight, hearing and other functions. Simple eyesight and hearing tests are often a first step, as conditions affecting these functions can often be detected at an early stage and treated. Sadly, this does not happen very often. But we could avoid a large burden of disability related to eyesight and hearing, if we screened for those conditions routinely at primary health care level.

Q: You mention the vast amount of scientific evidence on preventing the conditions associated with old age, to what extent is this evidence being used to inform public health policy and programmes?

A: It is happening, but not systematically. We need to change public health policy and practice. For example, we need to develop ambulatory care for older patients because if they are hospitalized they often lose physical and mental functioning. We should be targeting frail people in the community and intervening as early as possible to prevent their physical and mental deterioration. If we wait until a later stage, they will end up in a hospital emergency department or in long-term residential care. This is not an efficient way for health services to deal with such patients and, of course, it’s terrible for their quality of life. Most countries don’t do enough in this respect. General practitioners in some countries already give older people advice, for example, to take more physical exercise and improve their diet. But this must be done systematically at primary care level, based on current guidelines and other evidence-based recommendations and with the help of specialist services.

“We should be targeting frail people in the community and intervening as early as possible to prevent their physical and mental deterioration.”

Q: Do general practitioners have the diagnostic tools they need to intervene?

A: At the Gérontopôle, we developed a simple questionnaire for general practitioners to screen patients aged 65 years and over for frailty. After reviewing their findings for several frailty criteria, including recent tiredness and memory loss, the health professional makes a clinical judgement as to whether the patient is frail and at risk of further impairment or disability. We found that this questionnaire correctly identifies more than 95% of patients referred as frail or pre-frail. There are other simple tests too, for example, the seven-point Clinical Frailty Scale developed by Canadian specialists. Last year, the Frailty Consensus Conference in Florida, USA, recommended that people aged 70 years and older – as well as older people with 5% or more weight loss over the past year due to chronic illnesses – should be screened for frailty.

Q: How can countries like China and India rise to the challenge confronting them of increasing rates of frailty?

A: Most western countries in Europe and North America have waited too long to act and now we face an epidemic of severe dependency for daily activities – such as washing, dressing, going to the toilet, preparing food and eating – a dependency that is often irreversible. It is only now that public health professionals are starting to tackle these problems at an earlier stage. Countries such as China and India do not have to wait until people become totally dependent and end up sitting in a hospital bed. They can look out for frailty and intervene to prevent a large proportion of frailty now.

Q: A WHO Knowledge Network on Frailty is due to be launched next month, can you tell us about your collaboration with the World Health Organization (WHO) on this and its purpose?

A: We are working with WHO to implement frailty prevention in geriatric practice so that patients can maintain their autonomy. We are also working with a WHO expert group in collaboration with the International Association of Gerontology and Geriatrics on recommendations on how to develop and implement interventions in clinical practice to target frail people.

Q: Why has this area been neglected?

A: Geriatric medicine is still a new speciality and we are not listened to in the same way as experts from more established fields. Most governments have not anticipated the extent to which their populations are ageing. The solution is not a new drug – like statins for lowering cholesterol – and, therefore, this is not something that journals and medical conferences get excited about. However, this attitude is now changing and governments are realizing that the problem of dependency in older people can be extremely expensive and demoralizing for all concerned, considering the burden on families and social and health services.

Q: How can the forthcoming WHO Global Report on Ageing and Health address the need for prevention of conditions associated with old age?

A: Hopefully it will make governments and public health professionals more aware of the need to adapt current clinical practice to a rapidly ageing population to maintain their physical and cognitive functions as far as possible. This means maintaining mobility and memory and the ability to live at home rather than in institutions. Today’s medical practice employs highly sophisticated technologies to diagnose clinical problems but often forgets that patients need to maintain their basic functions and autonomy in everyday life. I hope that this report will convey the urgent message that we need to change clinical practice now, we cannot wait until it is too late and we have an epidemic of dependency.

